# Specific Oral Microbial Differences in Proteobacteria and Bacteroidetes Are Associated with Distinct Sites When Moving from Healthy Mucosa to Oral Dysplasia—A Microbiome and Gene Profiling Study and Focused Review

**DOI:** 10.3390/microorganisms11092250

**Published:** 2023-09-07

**Authors:** Allan Radaic, Eliah R. Shamir, Kyle Jones, Alessandro Villa, Nandita R. Garud, Aaron D. Tward, Pachiyappan Kamarajan, Yvonne L. Kapila

**Affiliations:** 1 School of Dentistry, University of California, Los Angeles (UCLA), Los Angeles, CA 90095, USA; aradaic@dentistry.ucla.edu (A.R.); pkamarajan@dentistry.ucla.edu (P.K.); 2 School of Dentistry, University of California, San Francisco (UCSF), San Francisco, CA 94143, USA; kylejonesdds@gmail.com (K.J.); alessandro.villa@baptisthealth.net (A.V.); 3 School of Medicine, University of California, San Francisco (UCSF), San Francisco, CA 94143, USA; eliah.shamir@ucsf.edu (E.R.S.); aaron.tward@ucsf.edu (A.D.T.); 4 Genentech, Inc., South San Francisco, CA 94080, USA; 5 Miami Cancer Institute, Baptist Health South Florida, Miami, FL 33176, USA; 6 College of Life Sciences, University of California, Los Angeles (UCLA), Los Angeles, CA 90095, USA; ngarud@ucla.edu; 7 Department of Human Genetics, David Geffen School of Medicine, University of California, Los Angeles (UCLA), Los Angeles, CA 90095, USA

**Keywords:** oral epithelial dysplasia, OED, OPMD, oral cancer, OSCC, microbial signature, Proteobacteria, Firmicutes, oral microbiome, dysbiosis

## Abstract

Oral potentially malignant disorders (OPMDs) are a group of conditions that carry a risk of oral squamous cell carcinoma (OSCC) development. Recent studies indicate that periodontal disease-associated pathogenic bacteria may play a role in the transition from healthy mucosa to dysplasia and to OSCC. Yet, the microbial signatures associated with the transition from healthy mucosa to dysplasia have not been established. To characterize oral microbial signatures at these different sites, we performed a 16S sequencing analysis of both oral swab and formalin-fixed, paraffin-embedded tissue (FFPE) samples. We collected oral swabs from healthy mucosa (from healthy patients), histologically normal mucosa adjacent to dysplasia, and low-grade oral dysplasia. Additionally, FFPE samples from histologically normal mucosa adjacent to OSCC, plus low grade and high-grade oral dysplasia samples were also collected. The collected data demonstrate significant differences in the alpha and beta microbial diversities of different sites in oral mucosa, dysplasia, and OSCC, as well as increased dissimilarities within these sites. We found that the Proteobacteria phyla abundance increased, concurrent with a progressive decrease in the Firmicutes phyla abundance, as well as altered levels of *Enterococcus cecorum*, *Fusobacterium periodonticum*, *Prevotella melaninogenica*, and *Fusobacterium canifelinum* when moving from healthy to diseased sites. Moreover, the swab sample analysis indicates that the oral microbiome may be altered in areas that are histologically normal, including in mucosa adjacent to dysplasia. Furthermore, trends in specific microbiome changes in oral swab samples preceded those in the tissues, signifying early detection opportunities for clinical diagnosis. In addition, we evaluated the gene expression profile of OSCC cells (HSC-3) infected with either *P. gingivalis*, *T. denticola*, *F. nucelatum*, or *S. sanguinis* and found that the three periodontopathogens enrich genetic processes related to cancer progression, including skin keratinization/cornification, while the commensal enriched processes related to RNA processing and adhesion. Finally, we reviewed the dysplasia microbiome literature and found a significant decrease in commensal bacteria, such as the *Streptococci* genus, and a simultaneous increase in pathogenic bacteria, mainly *Bacteroidetes* phyla and *Fusobacterium* genus. These findings suggest that features of the oral microbiome can serve as novel biomarkers for dysplasia and OSCC disease progression.

## 1. Introduction

Oral potentially malignant disorders (OPMDs) are a group of oral lesions that “carry a risk of cancer development in the oral cavity, whether in a clinically definable precursor lesion or in clinically normal mucosa” [1]. Oral dysplasia is the most common subset of OPMD [2,3], and risk factors include tobacco (either smoking and smokeless), betel quid nut, and alcohol use [4]. Dysplastic cells are characterized by hyperchromasia; enlargement of nuclei and, subsequently, decreased nuclear–cytoplasmic ratio; mitoses in suprabasal layers; loss of differentiation of keratinocytes towards the surface [5]; and, specifically for dysplasia of the upper aerodigestive tract, keratinization/cornification [6]. Based on histopathological grading of oral dysplasia, it is estimated that severe dysplasia has a malignant transformation rate of 7–50%, followed by moderate dysplasia (3–15%) and mild dysplasia (<5%) [7], with both moderate and severe dysplasia having a significant increased risk (OR 2.4 99% CI 1.5–3.8) of malignant transformation compared to mild dysplasia [8]. Despite these numbers, oral dysplasia grading does not reliably predict its clinical behavior and is by nature imprecise, with a high intra- and inter-observer variability in diagnosis [9,10], making it currently impossible to predict accurately which dysplastic lesions will progress to oral squamous cell carcinoma (OSCC) [11]. Thus, more accurate markers predicting oral dysplasia progression to cancer would enable better targeting of these lesions for closer follow-up, especially in the early stages of the disease [11]. Importantly, recent studies indicate that periodontal disease-associated pathogenic bacteria may have a role in neoplastic progression [12].

The human oral cavity harbors a complex and dynamic array of over 1000 “core” and “variant” microbial taxa that together constitute the oral microbiome [13,14]. Recent evidence from our group and others indicates that the oral microbiome, particularly its bacteria, plays a critical role in oral cancer pathogenesis [12,13,15,16,17,18,19,20,21,22]. Although the oral microbiota has evolved commensally to protect humans against foreign pathogens, its community becomes imbalanced (“dysbiotic”) throughout a person’s lifetime due to genetic risk factors and lifestyle behaviors, such as dietary intake, tobacco and alcohol use, and poor dental hygiene, thus pre-disposing the individual to oral pathology [23,24]. Dysbiosis is often characterized by a combination of reduced overall microbial diversity and negative changes in the relative abundances of beneficial and pathogenic microbes/bacteria [13,25]. Thus far, studies have demonstrated for dysplasia, in particular, a significant decrease in commensal bacteria, such as the *Streptococci* genus, and an simultaneous increase in pathogenic bacteria, mainly *Bacteroidetes* phyla and the *Fusobacterium* genus [26,27]. Moreover, epidemiologic studies have further demonstrated an association between periodontal disease/periodontal pathogens and oral and orodigestive cancers [15,28,29,30]. These changes suggest that the oral microbiome could have diagnostic or therapeutic potential for OSCC management, although it has not yet been explored for these applications.

In addition, it has not yet been established whether areas of histologically “normal” mucosa in patients with dysplasia have an oral microbiome composition more closely related to healthy mucosa (i.e., without dysplasia) or to dysplasia. Most studies in the field compare the oral microbiome of oral dysplasia tissues to either histologically normal adjacent/contralateral tissues or to whole mouth rinses or saliva from healthy patients [31,32,33,34,35,36]. Moreover, a recent meta-analysis specifically on oral dysplasia [26] indicates a high-risk of bias due to non-negligible heterogeneity of specimen types.

Understanding the potential microbial biomarkers involved during the transition from healthy mucosa to oral premalignant lesions (more specifically dysplasia) and to malignancy will be important for identifying novel diagnostic and therapeutic targets and ultimately improving oral cancer outcomes. Although the oral microbiome and its dysbiosis have been implicated in the pathogenesis of oral cancer, the microbial signatures associated with the transition from health to carcinogenesis have not been established [15,16,30,35,36,37,38,39,40,41,42,43]. Therefore, the objective of this pilot study was to determine the oral microbial signatures associated with healthy oral mucosa, oral dysplasia, and oral cancer.

## 2. Materials and Methods

### 2.1. Ethics Statement

Approval to conduct human subjects’ research, including protocols for the collection and use of human tissues (IRB# 19-29366; Reference #284015) and oral swab samples (IRB# 14-15342 and CC#15205 SPORE (Specialized Program of Research Excellence) in Head and Neck Cancer) were obtained from the University of California, San Francisco (UCSF) Institutional Review Board (IRB). Per IRB guidelines, patient consent was not required for archival (previously collected) formalin-fixed, paraffin-embedded (FFPE) specimens, but it was obtained for the collection of oral swab samples.

### 2.2. Inclusion Criteria

The inclusion criteria encompassed adults aged 18 or older with a biopsy-proven diagnose of oral dysplasia.

### 2.3. Oral Swab Sample Collection

Oral swab samples were collected in 2021 from 4 patients from areas of histologically normal oral mucosa adjacent to oral dysplasia and from the site of biopsy-proven low-grade oral dysplasia. Additionally, samples of histologically normal/healthy oral mucosa from the mandibular gingiva were collected from 4 patients with no history of oral dysplasia or OSCC as a control group. A total of 8 samples were collected by swabbing 10 times in a repeated motion over the mucosa with sterile cotton swabs. After collection, all swabs were immediately placed in RNA stabilization solution (RNALater, Millipore-Sigma, St. Louis, MO, USA) and stored at −80 °C until further processing.

### 2.4. Tissue Sample Collection

Samples from histologically normal oral mucosa adjacent to OSCC (*n* = 8), oral dysplasia (*n* = 13), and OSCC (*n* = 8), both matched and unmatched, were collected between 1999 and 2017 according to the approved protocol. All samples were from patients seen at UCSF for clinically detectable oral cavity lesions or cancer and were derived from archival FFPE tissue blocks collected for non-research purposes (medical treatment or diagnosis) at the UCSF Departments of Pathology and Oral Pathology.

### 2.5. DNA Extraction

Tissue samples were deparaffinized using the QIAamp DNA FFPE Tissue Kit (Qiagen, Germantown, MA, USA), according to the manufacturer’s instructions. Total DNA was extracted from both tissue and swab samples using a QIAamp DNA Mini Kit (Qiagen, USA), according to the manufacturer’s instructions. Next, the total DNA content for each sample and its overall quality was assessed using a Nanodrop One UV-Vis Spectrophotometer (ThermoFisher Scientific, Waltham, MA, USA), and the DNA samples were kept at −80 °C until 16S sequencing (Novogene Corp Inc., Sacramento, CA, USA). Insufficient DNA was recovered for sequencing from 2 of the histologically normal adjacent FFPE specimens and 1 of the dysplasia swab samples.

### 2.6. 16S Amplification and Sequencing

The DNA purity of the samples was first monitored with 1% agarose gels using DNA diluted to 1 ng/μL in sterile water. Then, the 16S rRNA genes of the V4 region were amplified using 515F-806R primers and Phusion High-Fidelity PCR Master Mix (New England Biolabs, Ipswich, MA, USA). Next, the PCR products were mixed (1:1 *v*/*v*) with 1X loading buffer (containing SYBR green) and loaded onto a 2% agarose gel for electrophoresis detection. Samples with bright single bands between 400 and 450 bp were chosen and purified with a Qiagen Gel Extraction Kit (Qiagen, Hilden, Germany) for further analysis. Finally, the 16S libraries were generated using the NEB Next Ultra DNA Library Preparation Kit (New England Biolabs, USA) and analyzed via Illumina NovaSeq 6000 platform by Novogene Corp Inc. (Sacramento, CA, USA).

### 2.7. Sequencing Data Processing

Paired-end reads were merged via FLASH [44], a fast and accurate analysis tool designed to merge overlapping paired-end reads (raw tags). Quality filtering on the raw tags was performed under specific filtering conditions to obtain high-quality clean tags [45] using QIIME’s quality-controlled process [46]. Next, the raw tags were compared to the SILVA reference database via the UCHIME algorithm [47] to detect and remove chimera sequences [48], thereby obtaining the effective tags.

### 2.8. Operational Taxonomic Unit (OTU) Cluster and Taxonomic Annotation

All 16S effective tags were analyzed using UPARSE software (v. 7.0.1090) [49]. Sequences with ≥97% similarity were assigned to the same OTUs, obtaining the representative sequences. Each representative sequence was then compared against the SSUrRNA database of the latest version of SILVA Database [50] at each taxonomic rank (i.e., kingdom, phylum, class, order, family, genus, and species) using a threshold of 0.8–1 [51] via QIIME [46]. Then, MUSCLE [52] was used to obtain the phylogenetic relationship of all OTUs’ representative sequences. Finally, all the OTUs’ abundances were normalized using a standard of sequence number corresponding to the sample with the least sequences. Subsequent analyses of alpha diversity and beta diversity were all performed based on this generated normalized data.

### 2.9. Alpha Diversity

Alpha diversity indices represent the diversity of species in an ecosystem, summarizing the structure of that particular ecological community. Many perturbations can affect a community’s alpha diversity, especially in microbial communities. Thus, comparing community structure via alpha diversity is an initial step to analyze how the microbial community changes under different conditions. To do this, several metrics are used to establish the community richness (i.e., the number of taxonomic groups in the samples), its evenness (i.e., the distribution of taxonomic groups within the community), or both [53].

Three indices of alpha diversity were computed in this study: the observed number of species (i.e., the count of unique OTUs found in the sample, estimating its richness), the Shannon diversity (which estimates both species richness and evenness), and the Chao1 diversity (which estimates total species richness in the sample). All indices were calculated using the QIIME Software [54].

### 2.10. Beta Diversity

While alpha diversity is the representation of species in a particular ecosystem, beta diversity is the measure of the differences in species composition between two or more local communities or even between local and regional ecosystems [55,56]. In this study, the beta diversity was measured via unweighted UniFrac with the QIIME software [54]. Then, principal coordinate analysis (PCoA) was performed on UniFrac estimates to visualize complex and multidimensional data. Unweighted Pair-group Method with Arithmetic Means (UPGMA) Clustering was performed as a type of hierarchical clustering method to interpret the distance matrix using average linkage and was conducted with QIIME software [54].

### 2.11. HSC-3 Cell Culture

Oral cancer (HSC-3) cell line was maintained as previously described [17,57,58]. Briefly, cells were grown in DMEM medium supplemented with 10% FBS and 1% penicillin and streptomycin under a humid atmosphere at 37 °C and 5% CO_2_. Cells were subcultured with trypsin/PBS every 2 or 3 days.

### 2.12. Bacterial Culture

*Treponema denticola* (ATCC 35405), *Porphyromonas gingivalis* (FDC 381), *Fusobacterium nucleatum* (ATCC 10953), and *Streptococcus sanguinis* (S160) were anaerobically grown as described previously [59,60,61,62]. Briefly, *T. denticola* was cultured in Oral Treponeme Enrichment Broth (OTEB); *P. gingivalis* and *F. nucleatum* were culture in Brain Heart Infusion (BHI) broth supplemented with 5 μg/mL of hemin and 1 μg/mL vitamin K; and *S. sanguinis* was cultivated using plain BHI broth. Anaerobic conditions were obtained by placing bacterial samples into sealed anaerobic jars that underwent five cycles of depressurization (vacuum formation) and nitrogen (N_2_) pressurization (1 ATM) and kept at 37 °C in an Isotemp Incubator (Thermo-Fisher Scientific, USA). The bacteria were split every 2–3 days (except for *T. denticola*, which was split every 7 days).

### 2.13. RNAseq

HSC-3 cells (10^6^ cells per plate) were plated on 60 mm cell culture dishes (Corning, Corning, NY, USA) and let adhere overnight. On the next day, cells were infected with either *P. gingivalis*, *T. denticola*, *F. nucleatum*, or *S. sanguinis* (at 50 MOI) and incubated for 2 h. Then, the cells were washed with PBS three times for bacterial removal and incubated for another 24 h after the addition of fresh media. Finally, their RNA was extracted using RNeasy mini kit (Qiagen, USA) and samples were submitted to Novogene Corp Inc. (Sacramento, CA, USA) for RNAseq.

### 2.14. Statistical Analyses

Parametric statistical analyses were performed via GraphPad Prism (v. 10, San Diego, CA, USA), whereas non-parametric analyses were performed via R Software (v. 2.15.3, USA). OTU (i.e., phyla, class, family, and genus), as well as alpha diversity rarefaction are reported as means ± SD, and their statistical analyses were performed via Two-Way ANOVA, as described in their respective figure legends. Alpha and beta diversity indices are reported as medians ± min and max, and their statistical analyses were performed using Wilcoxon signed-rank test.

## 3. Results

### 3.1. Demographics of Study Patients

Oral swab samples were collected from four patients from the surface of oral leukoplakia lesions with histologic evidence of oral epithelial dysplasia. For matched internal controls, oral swab samples were collected from the surface of clinically normal appearing tissue adjacent to the oral dysplastic lesions. As additional controls, we collected oral swab samples from the mandibular gingiva of four healthy subjects with no history of oral dysplasia or OSCC and with no other oral mucosal lesions. The demographics for these groups are summarized in Table 1, and no significant differences were found between groups.

In addition to the oral swabs, 30 archival FFPE tissue specimens of histologically normal mucosa adjacent to OSCC, oral dysplasia, and OSCC were obtained from 17 patients. The demographics for these patients are summarized in Table 2. Dysplasia samples were subdivided into low-grade (mild or moderate dysplasia) or high-grade (severe dysplasia or squamous cell carcinoma in situ (SCCIS)). Samples were analyzed in two separate cohorts. The first cohort consisted of 13 specimens from 11 patients, including 2 samples of histologically normal mucosa adjacent to OSCC, 7 low-grade dysplasia, and 4 high-grade dysplasia. One patient in this group had two synchronous biopsies demonstrating different dysplasia grades, and a second patient had two biopsies showing low-grade dysplasia collected three years apart. The second cohort consisted of six patients with matched samples from the same resection specimen: four patients had matched histologically normal adjacent, low-grade dysplasia, and OSCC; one patient had matched histologically normal adjacent, low-grade dysplasia, and high-grade dysplasia; and one patient had matched histologically normal adjacent and high-grade dysplasia with possible microinvasion. HPV testing was not performed on all these cases, as only a small fraction (<4%) of OSCC has been reported as HPV-positive [63,64] and the specific lesions tested did not show the typical HPV basaloid histomorphology. No significant demographic differences were found across groups.

16S sequencing of all the samples yielded an average of 148,446 reads per sample, totaling 5,640,949 pre-processing sequences. For two of the histologically normal adjacent and one of the low-grade dysplasia swab samples, insufficient DNA was recovered for sequencing. These sequences were binned into a total of 9158 OTUs.

### 3.2. Oral Dysplasia and OSCC Microbiome Communities Are Distinct from Those in Healthy and Histologically Normal Adjacent Communities

**Figure 1:**
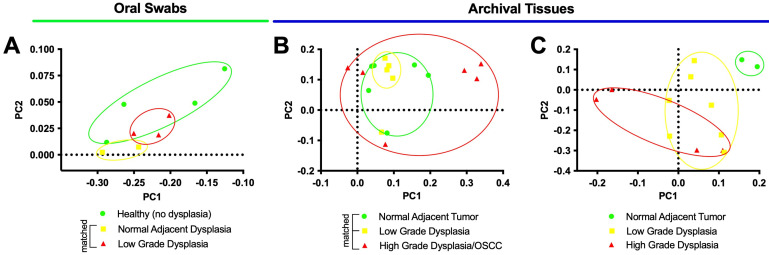
Microbiome communities from oral dysplasia and OSCC have distinct profiles compared to healthy and histologically normal adjacent communities. Principal coordinate analysis (PCoA) for oral swab samples (**A**), matched tissue samples (**B**), and unmatched tissue samples (**C**). Circles indicate the 95% confidence interval (CI 95%) for each group.

We started by analyzing the microbial communities via principal coordinate analysis (PCoA). Figure 1 shows the results of the three groups of samples—oral swabs (Figure 1A), matched FFPE tissues (Figure 1B), and unmatched FFPE tissues (Figure 1C).

In PCoA, sample groups that are closer to one another are more similar than those positioned further away [56]. In the current study overall, the sample groups exhibited areas of ellipse overlap and therefore similarity, especially when analyzing the matched samples (Figure 1B). For example, the low-grade dysplasia specimens were more similar to the histologically normal adjacent specimens than to high-grade dysplasia/OSCC (Figure 1B). Regarding the oral swab samples (Figure 1A), a larger degree of dissimilarity was observed amongst the healthy (no dysplasia) samples, whereas the histologically normal adjacent and the dysplasia communities had less variance across patients. The dysplasia community partially overlapped as a subset within the healthy community. Finally, unmatched tissue samples (Figure 1C) indicate comparable dissimilarities between low- and high-grade dysplasia compared to histologically normal adjacent control.

Additionally, ellipsis overlap can also indicate dissimilarity among sample groups they are not overlapping with other sample group’s ellipses. In the current study, among the unmatched samples (Figure 1C), both low-grade and high-grade dysplasia specimens’ ellipses were distinct (i.e., have no overlap) to histologically normal adjacent specimens and, therefore, dissimilar to it. In the current study, this dissimilarity to histologically normal adjacent control can indicate that both low- and high-grade dysplasia may be associated with distinct microbiomes, compared to the histologically normal adjacent tissues [65].

**Figure 2:**
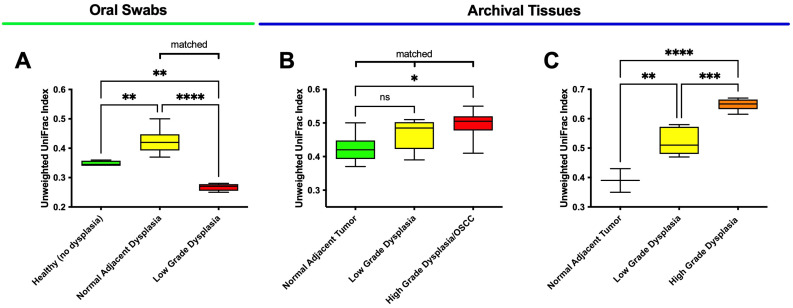
Beta diversity reveals different community composition in healthy versus dysplasia specimens. Unweighted UniFrac beta diversity for oral swab samples (**A**), matched tissue samples (**B**), and unmatched tissue samples (**C**). ns means not significant; * means *p* ≤ 0.05; ** means *p* ≤ 0.01; *** means *p* ≤ 0.001 and **** means *p* ≤ 0.0001 between marked samples.

Beta diversity measures the total effective number of species in a group of samples divided by the effective number of species in each sample [56]. The results for the three groups can be seen in Figure 2.

Beta diversity among oral swab samples (Figure 2A) showed that histologically normal adjacent to dysplasia specimens were significantly different from low-grade dysplasia (*p* < 0.0001) and from healthy mucosa from other patients (*p* = 0.007). Additionally, low-grade dysplasia specimens showed a significant lower UniFrac Index (*p* = 0.008) compared to healthy mucosa (no dysplasia). In contrast, among matched FFPE tissues (Figure 2B), low-grade dysplasia showed no significant differences from histologically normal adjacent mucosa (*p* = 0.080) despite showing a trend toward higher levels, and the only significant difference was between OSCC and histologically normal adjacent samples (*p* = 0.015). Among the unmatched samples (Figure 2C), beta diversity in both low- and high-grade dysplasia was significantly higher (*p* = 0.004; *p* < 0.0001, respectively) compared to histologically normal adjacent to tumor specimens. Furthermore, the beta diversity of high-grade dysplasia was also found to be significantly higher compared to low-grade dysplasia (*p* = 0.0003).

In addition to the PCoA results, the beta diversity results provide an additional level of information for evaluating dysplasia (i.e., for distinguishing between low-grade and high-grade dysplasia samples) that may be helpful for elucidating the microbiome shifts underlying transitions from health to dysplasia and cancer.

As previously described, our oral swab data further showed that the beta diversity of the “healthy” (no dysplasia) microbiome is significantly different from that of histologically normal adjacent to dysplasia specimens, indicating that microbiome shifts may have taken place in the histologically normal adjacent tissues. This highlights the potential need for future studies to evaluate the microbiome of healthy tissues (with no history of oral dysplasia or OSCC) as a control group, in addition to histologically normal adjacent tissues.

### 3.3. High-Grade Dysplasia and OSCC Alpha Diversities Are Significantly Different from Those in Histologically Normal Adjacent Specimens

**Figure 3:**
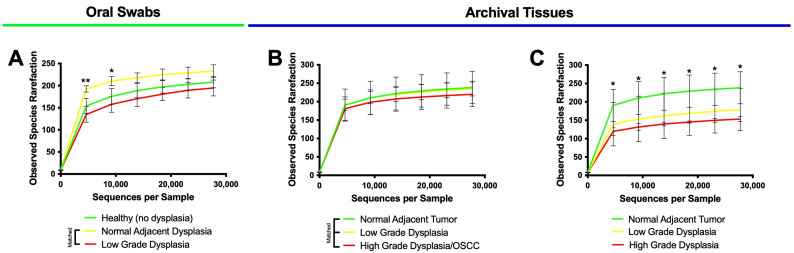
Rarefaction curves for all samples converge into a horizontal asymptote. Observed species rarefaction for oral swab samples (**A**), matched tissue samples (**B**), and unmatched tissue samples (**C**). * means *p* ≤ 0.05; ** means *p* ≤ 0.01 between healthy (no dysplasia) and histologically normal adjacent to dysplasia.

Alpha diversity summarizes the structure of an ecological community in the context of its richness (i.e., the number of observed species) and/or evenness (i.e., the distribution of abundances of the species) [53]. As an initial step, we rarefied the observed species, and the results can be seen in Figure 3.

**Figure 4:**
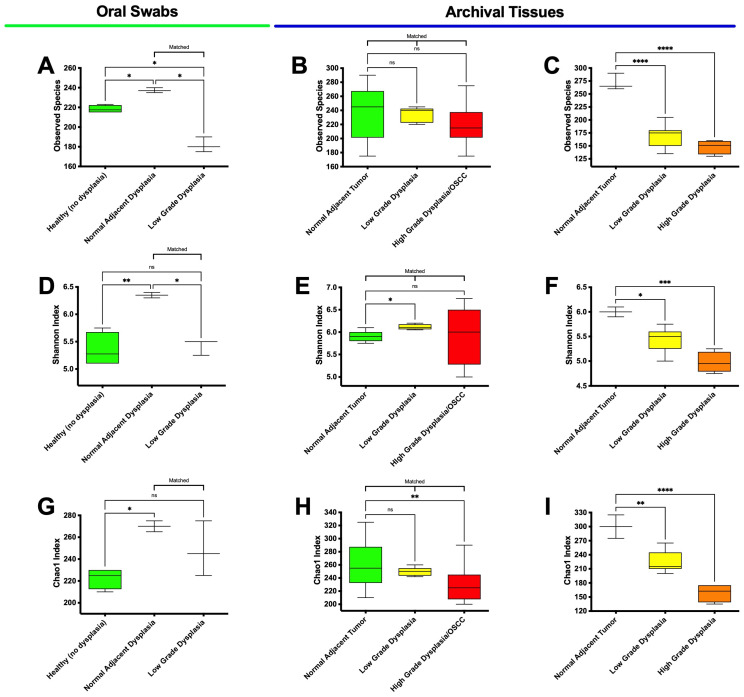
Alpha diversity in high-grade dysplasia is significantly different from that in histologically normal adjacent to tumor specimens. Alpha diversity assessed via observed species (panels (**A**–**C**)), Shannon Indices (**D**–**F**), and Chao1 Indices (**G**–**I**) for the oral swab samples (**A**,**D**,**G**), matched tissue samples (**B**,**E**,**H**), and unmatched tissue samples (**C**,**F**,**I**). ns means not significant; * means *p* ≤ 0.05; ** means *p* ≤ 0.01; *** means *p* ≤ 0.001; and **** means *p* ≤ 0.0001 between marked samples.

The results showed that all rarefaction curves converged into a horizontal asymptote, indicating that further observations (i.e., more sequence reads) would have little or no effect on the observed species metrics subsequently analyzed. Thus, we proceeded to evaluate alpha diversity. The results for the observed species and the Shannon and Chao1 Indices can be seen in Figure 4.

Regarding the observed species in the oral swab samples (Figure 4A), the results showed significant differences between low-grade dysplasia and healthy (no dysplasia) (*p* = 0.012), as well as compared to clinically normal adjacent to dysplasia samples (*p* = 0.011). Significant differences between histologically normal (no dysplasia) and histologically normal adjacent to dysplasia was also found (*p* = 0.029). In the matched FFPE tissues samples (Figure 4B), a downward trend was seen in the mean observed species from the clinically normal adjacent to tumor to low-grade dysplasia and high-grade dysplasia/OSCC samples, although no statistically significant difference was found. Within the unmatched FFPE tissues (Figure 4C), high-grade dysplasia (*p* < 0.0001) and low-grade dysplasia (*p* = 0.001) were both significantly different from the histologically normal adjacent specimens.

An analysis using the classic alpha diversity index—the Shannon Index (Figure 4D–F)—showed that diversity in oral swabs (Figure 4D) was significantly increased in histologically normal adjacent to dysplasia samples, compared to both healthy (no dysplasia) (*p* = 0.07) and matched low-grade dysplasia (*p* = 0.012) specimens. The data also showed a significant difference in low-grade dysplasia compared to histologically normal adjacent to tumor for both matched (Figure 4E—*p* = 0.011) and unmatched tissue samples (Figure 4F—*p* = 0.020), although in opposing directions. Moreover, the unmatched tissue samples (Figure 4F) also indicated a significant decrease in diversity for high-grade dysplasia (*p* = 0.001) compared to histologically normal adjacent to tumor specimens. No significant differences were found between low-grade and high-grade dysplasia for both matches (Figure 4E) and unmatched (Figure 4F) tissue samples.

Finally, the non-parametric Chao1 index (Figure 4G) for the oral swab specimens indicated a significant difference (*p* = 0.027) between histologically normal adjacent to dysplasia and healthy (no dysplasia) specimens, while no significant differences were found between low-grade dysplasia and histologically normal adjacent to dysplasia. In the matched tissues (Figure 4H), there was a significant decrease in the high-grade dysplasia/OSCC (*p* = 0.002) compared to histologically normal adjacent to tumor samples, while no significant differences were found between low- and high-grade dysplasia. Corroborating the data of the other two indexes, the Chao1 data for the unmatched specimens (Figure 4I) also indicated a significant decrease for low- and high-grade dysplasia specimens (*p* = 0.004 and *p* < 0.001, respectively) compared to the histologically normal adjacent to tumor specimens. Additionally, our results showed that the observed species in the histologically normal adjacent oral swab samples were not significantly different from those found in the histologically normal adjacent matched (*p* = 0.8959) and unmatched (*p* = 0.8827) tissue samples (Supplemental Materials, Figure S1), validating what other studies have found thus far [66]. Moreover, Villa, and Gohel [67] have shown that out of more than 3100 patients screened, only 27 (0.9%) of them presented with OPMDs and only 3 (0.09%) specifically exhibits dysplasia. Thus, sampling the oral microbiome via oral swabs may be an useful method for capturing microbiome data comparable to tissue sampling, especially in hard-to-obtain samples, such as OPMDs and oral dysplasia tissues.

### 3.4. Significant Increases in Proteobacteria and Decreases in Firmicutes as well as Expansion of Fusobacteria Are Noted When Moving from the Clinically/Histologically Normal Oral Mucosa to Dysplasia and to Cancer

**Figure 5:**
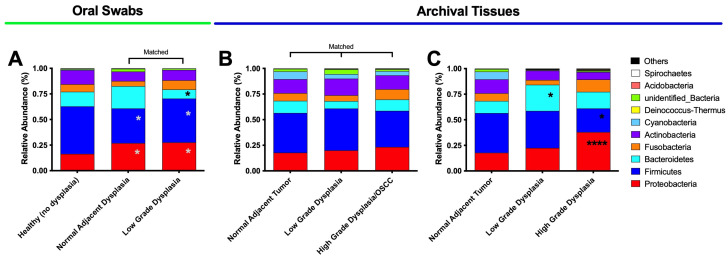
A significant increase in Proteobacteria and a decrease in Firmicutes phyla as well as expansion of Fusobacteria characterized the changes from health to disease (dysplasia and OSCC). Phylum relative abundance for oral swab samples (**A**), matched tissue (**B**), and unmatched tissue samples (**C**). * means *p* ≤ 0.05 between the marked sample and histologically normal adjacent dysplasia; **** means *p* ≤ 0.0001 between the marked sample and histologically normal adjacent to tumor; and * means *p* ≤ 0.05 between the marked sample and healthy (no dysplasia).

Next, we analyzed the relative abundance of the top 10 taxa in the oral swab and FFPE tissue samples at the phylum and class levels (Figure 5).

The results for the phyla analysis for the oral swab samples (Figure 5A) revealed a significant increase in the Proteobacteria phyla (*p* = 0.025; *p* = 0.047) and a decrease in the Firmicutes phyla (*p* = 0.047; *p* = 0.028) in both the low-grade dysplasia and histologically normal adjacent to dysplasia samples, respectively, compared to healthy (no dysplasia) samples. A similar trend was observed in the high-grade dysplasia in the unmatched tissues (Figure 5C)—a significant increase in Proteobacteria (*p* < 0.0001) and significant decrease in Firmicutes (*p* = 0.0184) phyla compared to histologically normal adjacent samples. In the oral swab samples, we further found a non-significant tendency of increased Bacteroidetes from healthy normal to histologically normal adjacent, followed by significant decrease in the low-grade dysplasia samples compared to matched histologically normal adjacent specimens (*p* = 0.032) (Figure 5A). However, in the unmatched tissue samples, the low-grade dysplasia specimens (Figure 5C) showed a significant increase in Bacteriodetes phyla (*p* = 0.041) and a non-significant tendency to decrease on high grade dysplasia. Additionally, a significant decrease in the Firmicutes phyla (*p* = 0.0119) compared to histologically normal adjacent to tumor specimens were also detected. No significant differences were found among the matched FFPE tissue samples (Figure 5B).

**Figure 6:**
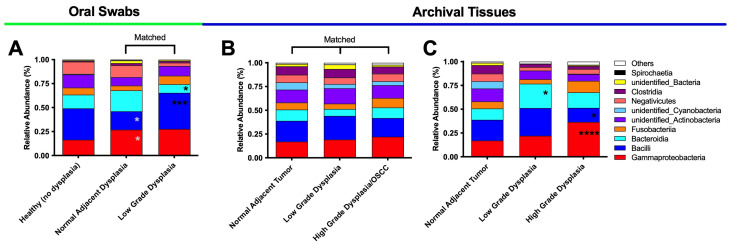
Significant increases in Gammaproteobacteria and decreases in Bacilli classes characterized the changes when moving from histologically normal oral mucosa to dysplasia to OSCC. Class relative abundance for oral swab samples (**A**), matched tissue samples (**B**) and unmatched tissue samples (**C**). * means *p* ≤ 0.05; *** means *p* ≤ 0.001; **** means *p* ≤ 0.0001 between the marked sample and histologically normal adjacent; and * means *p* ≤ 0.05 between the marked sample and healthy (no dysplasia).

Next, we evaluated the class analysis for the swab samples and tissue samples (Figure 6).

For oral swab samples (Figure 6A), the relative abundance data revealed a significant increase in the Bacilli class (*p* = 0.0010) and a significant decrease in the Bacteroidia class (*p* = 0.0284) in the low-grade dysplasia compared to their matched histologically normal adjacent to dysplasia samples. The oral swab samples also indicated a significant increase in the Gammaproteobacteria class (*p* = 0.0213) and a significant decrease in the Bacilli class (*p* = 0.0134) in the histologically normal adjacent samples compared to their relative abundance in the healthy (no dysplasia) samples.

For the unmatched tissue samples (Figure 6C), the data indicated a significant increase in the Gammaproteobacteria (*p* < 0.0001) and a significant decrease in the Bacilli (*p* = 0.0335) classes in the high-grade dysplasia/OSCC, compared to histologically normal adjacent to tumor. Contrary to the swab samples, a significant expansion of the Bacteroidia class was seen in the low-grade dysplasia, compared to histologically normal adjacent to tumor specimens (Figure 6C). No significant differences were found among the matched tissue samples (Figure 6B), although there was a tendency toward increased levels of Fusobacteriia classes and decreased levels of Bacilli in the high-grade dysplasia compared to the low-grade dysplasia. There was also a tendency toward decreased levels in Bacteroidia, unidentified Cyanobacteria and Fusobacteriia classes, and increased levels of Bacilli and unidentified Actinobacteria classes in the low-grade dysplasia compared to histologically normal adjacent to tumor.

In family OTU analysis (Figure S2) for the oral swab samples, we found the *Pasteurellaceae* (*p* = 0.024) family to be significantly increased in the low-grade dysplasia compared to the healthy (no dysplasia) specimens. On the other hand, in the unmatched tissue samples, we found the *Burkoholdericeae* (*p* = 0.0013) family significantly increased in the high-grade dysplasia specimens compared to histologically normal adjacent to tumor specimens (Figure S2C). Notably, the most dominant family in the unmatched tissue samples (i.e., *Burkholderiaceae* family) was missing in the oral swab samples (Figure S2).

In the genus OTU analysis (Figure S3), we found the *Streptococci* genus (*p* = 0.0065) to be significantly reduced in the histologically normal adjacent to dysplasia specimens compared to healthy (no dysplasia) oral swab samples (Figure S3A). Combined with the phyla level data, these results reiterate the possibility that the histologically normal adjacent samples may exhibit a shift in their microbiome composition compared to healthy tissues.

**Figure 7:**
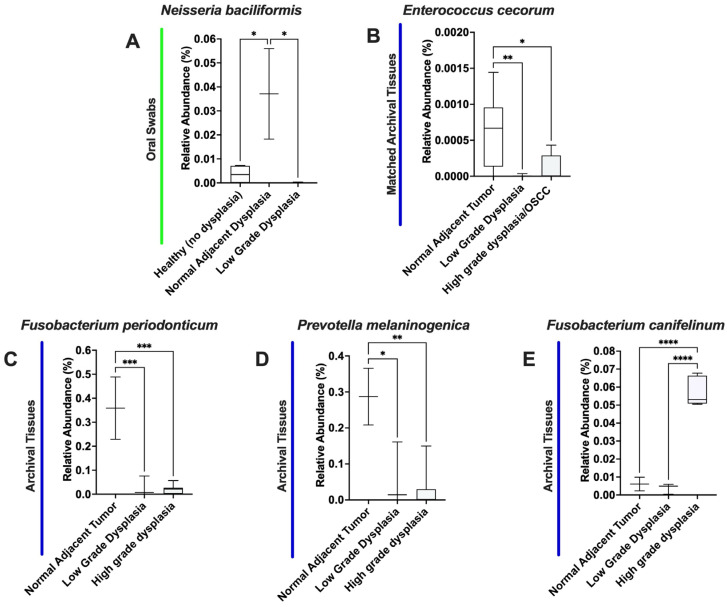
*Neisseria baciliformis* (**A**), *Enterococcus cecorum* (**B**), *Fusobacterium periodonticum* (**C**), *Prevotella melaninogenica* (**D**), and *Fusobacterium canifelinum* (**E**) are significantly different when moving from histologically normal to low- and high-grade dysplasia. * means *p* ≤ 0.05; ** means *p* ≤ 0.01; *** means *p* ≤ 0.001; and **** means *p* ≤ 0.0001 between the marked samples.

In species OTU analysis (Figure 7), we found five bacterial species with significantly different abundances in different sites. For swab samples, *Neisseria baciliformis* was found to be significantly elevated in histologically normal adjacent to dysplasia compared to both healthy (no dysplasia) (*p* = 0.318) and low-grade dysplasia (*p* = 0.0265). For the matched tissue samples, *Enterococcus cecorum* was found to be significantly lower in low grade dysplasia (*p* = 0.0092) and high-grade dysplasia/OSCC (*p* = 0.0371). For the unmatched tissues, *Fusobacterium periodonticum* and *Prevotella melaninogenica* was significantly lower in both low-grade (*p* = 0.0007 and *p* = 0.0118) and high-grade dysplasia (*p* = 0.0002 and *p* = 0.0025) compared to histologically normal adjacent to tumor sites, while *Fusobacterium canifelinum* was found to be significantly higher compared to both histologically normal adjacent to tumor (*p* < 0.0001) and low-grade dysplasia (*p* < 0.0001).

**Figure 8:**
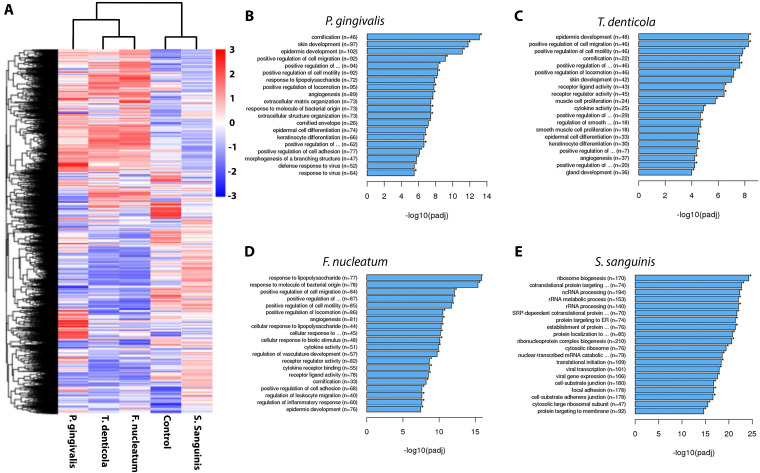
*P. gingivalis*, *T. denticola*, and *F. nucleatum* significantly shifts the gene expression profile of the host cell (HSC-3 cell line) compared to *S. sanguinis* and the control. (**A**) Gene expression profile heatmaps of HSC-3 cells exposed to *P. gingivalis*, *T. denticola*, *F. nucleatum*, and *S. sanguinis*; Gene ontology enrichment analysis for (**B**) *P. gingivalis*, (**C**) *T. denticola*, (**D**) *F. nucleatum*, and (**E**) *S. sanguinis*. * means processes significantly different (*p* < 0.05) compared to control.

Finally, we evaluated the effects of four bacterial species on the gene expression of an OSCC cell line (HSC-3). Two of these species (i.e., *Fusobacterium nucleatum* and *Porphyromonas gingivalis*) were identified as significantly increased in dysplasia and OSCC in the focused literature review (Section 4): one species which has been recently specifically implicated with development and progression of OSCC (i.e., *Treponema denticola*) and one species (i.e., *Streptococcus sanguinis*) representing *Streptococcus* genus, which has been identified as significantly decreased in dysplasia and OSCC (by both current results and literature reviews) [12,17,57,68]. The results (Figure 8) show that the three periopathogens (i.e., *P. gingivalis*, *T. denticola*, and *F. nucleatum*) significantly changed the gene expression profile compared to the control and *S. sanguinis* (Figure 8A). Next, we performed a gene ontology enrichment profile on the data (Figure 8B–E). The three periopathogens significantly enriched processes related to cancer progression, such as positive regulation of cell migration and cell motility, angiogenesis, regulation of vasculature development, regulation of leukocyte migration, and cytokine activity processes compared to the control. Additionally, these pathogens also enriched processes related to the cell keratinization and differentiation, such as skin and epidermis development, epidermal and keratinocyte differentiation, as well as cornification and cornified envelope processes. On the other hand, *S. sanguinis* significantly enriched processes related to the ribosome, such as ribosome biogenesis, non-coding RNA (ncRNA), and rRNA metabolic processing, as well as significantly enriched focal adhesion and cell–substrate junction processes compared to the control.

## 4. Discussion

Oral dysplasia is defined as a lesion in which part of the lining mucosa shows varying degrees of cellular atypia, maturation, and differentiation disturbances [4,69]. Tobacco (either smoking and smokeless), betel quid nut, and alcohol use are known risk factors for oral dysplasia [4], and therefore, preventive measures should include avoiding tobacco and betel quid nut use and limiting alcohol intake.

To date, very few studies have specifically examined the microbiome signature in dysplasia tissues (Table 3). The earliest study we found (Krogh et al. [70] from 1987) analyzed *Candida* species infecting OPMD biopsies with and without dysplasia using basic yeast culture. Out of 12 OPMD samples, only 5 (41.6%) were diagnosed with any degree of dysplasia. Out of those five cases, the authors found five different strains of *Candida albicans*, one strain of *Candida parapsilosis*. Interestingly, *Candida* strains found in dysplasia-positive samples had lower nitrosation rate compared to the ones on dysplasia-negative samples, although no statistical analysis was performed. This, however, may indicate that other species, such as *Phorphyromonas gingivalis,* may be responsible for the production of *N*-nitroso compounds, which have been associated with increased risk of OSCC [71].

In a recent study (2023), Shen et al. [26] systematically reviewed the literature on the oral microbiome in dysplasia tissues and found that the analyzed studies presented a high risk of bias due to non-negligible heterogeneity in the type and size of the sample and inconsistent oral microbiome composition, strongly limiting the analysis. However, out of the 11 selected studies, only 6 of those histopathologically diagnosed dysplasia in the tissues, 1 of which (Herreros-Pomares et al. [73]) did not control for dysplasia and mixed the non-dysplasia samples with mild, moderate, and severe dysplasia samples. These discrepancies, nevertheless, may have accounted for the non-negligible inconsistencies and heterogeneity found in the analysis.

Overall, those studies consistently found a significant decrease in commensal bacteria, such as the *Streptococci* genus, and a simultaneous increase in pathogenic bacteria, mainly Bacteroidetes phyla, and the *Phorphyromonas* and *Fusobacterium* genera. Additionally, the literature also points out that this signature seems to follow a healthy → dysplasia → OSCC progression [27], where less of the commensal and more of the pathogenic bacteria are found in OSCC, compared to leukoplakia/dysplasia, highlighting that oral microbiome shifts in dysplasia may be related to disease progression. Interestingly, a lack of significant changes in malignant-progressing dysplasia microbiome compared to non-malignant progressing ones have been reported [72], suggesting that the initial microbiome that precedes the development of dysplasia may be more important in determining the fate of the tissue than that present once the dysplasia has been established. Another hypothesis is that the difference may actually stem from dysregulated molecular pathways in the dysplasia tissues triggered by the microbiome. Ganly et al. [27] reported significant increases in the *HSP90* gene and TLRs 1, 2, and 4 ligands along the progression from health to OSCC. Conway et al. [82] reported 167 differently expressed genes in dysplasia compared to healthy tissues, with a significant increase in immune response, leukocyte, and lymphocyte activation genes. Interestingly, these immune response genes were not significantly different when compared to OSCC. Similarly, Abdalla et al. [83] demonstrated a significant loss of plasma membrane expression of both *E-cadherin* and *EMP-1* in patient biopsies from oral dysplasia, which were similar to that seen in the T1 and T4 stages of OSCC. Taken together, these molecular changes in dysplasia suggest that both immune response and adhesion/epithelial pathways may underlie early carcinogenesis development in dysplasia. Therefore, more retrospective studies comparing the microbiome of malignant-progressing vs. non-malignant-progressing dysplasia tissues, more retrospective studies testing the microbiome in the transition state from health to dysplasia, and further molecular studies on these tissues are needed.

In this context, the objective of this study was to evaluate the changes in the oral microbiome signatures in the changes from health to dysplasia and to cancer. The transition of the oral mucosa to dysplasia is particularly important as it may represent the earliest stages in the disease process, and therefore, data from this transition step may be useful in defining early microbial mediators or regulators of subsequent cancer development.

We showed that specific microbial and community composition shifts were present when moving from histologically normal mucosa to dysplasia and to OSCC, indicating robust and distinct signals during these shifts toward disease. Specifically, we found significant differences in the alpha and beta diversities among healthy mucosa, histologically normal adjacent mucosa, low-grade dysplasia, and high-grade dysplasia/OSCC. Additionally, we found that Proteobacteria and Fusobacteria phyla abundance increased, concurrent with a decrease in the Firmicutes phyla abundance in the unmatched FFPE tissue changes/transitions, but not in the matched samples. We also found significant altered levels of *Enterococcus cecorum*, *Fusobacterium periodonticum*, and *Prevotella melaninogenica* and when moving from histologic normal to low- and high-grade dysplasia. *Fusobacterium canifelinum* levels were significantly higher in high grade dysplasia compared to both histologically normal adjacent and low-grade dysplasia.

Our data contribute to the current literature by highlighting that there are robust microbiome community changes present when moving from health to disease states and by demonstrating that there are also oral microbiome changes present in oral dysplasia (from both FFPE tissues and oral swab samples) relative to healthy or histologically normal adjacent mucosa. Moreover, our data further establish that sites with histological normal mucosa in patients with dysplasia or OSCC show microbial alterations compared to those of healthy sites with no history of oral dysplasia and OSCC. In fact, Babji et al. [84] has demonstrated significant histomorphometric changes in basal cells of histologically normal oral mucosa extracted from >1 cm away from OSCC compared to healthy oral mucosa of patients with no history of OSCC undergoing tooth extraction. Remarkably, we found significantly higher levels of *Neisseria baciliformis*, an opportunistic pathogen [85], in histological normal adjacent to dysplasia samples compared to patients with no history of oral dysplasia. This species can be used as a potential biomarker of future dysplastic transformation in healthy patients with no history of dysplasia or OSCC. Given these findings, we recommend the inclusion of sampling from healthy sites (with no history of oral dysplasia and OSCC) in future studies that examine the contributions of the microbiome to disease.

The possibility of pathogenic bacterial growth in cancers has been attributed to unique pathophysiologic features present in many cancers, which may benefit the growth of these particular bacteria, such as impaired and abnormal vascular architecture, an enhanced permeability and retention effect, low oxygen pressure/hypoxia, and extensive necrosis [12,86,87]. Specifically for OSCC, increased salivary bacterial counts of *Lactobacillus* species, *Capnocytophaga gingivalis*, *Prevotella melaninogenica*, and *Streptococcus mitis* and loss of *Haemophilus*, *Neisseria*, *Gemella*, and *Aggregatibacter* genera have been reported in oral cancer patients compared with matched histologically normal adjacent controls [30,36,87,88]. Our group previously identified a high *Fusobacterial* and low *Streptococcal* phenotype as part of the transition from health to primary and metastatic oral and head and neck cancer [16]. Additionally, a recent metadata analysis on oral epithelial dysplasia indicates increases in the Bacteroidetes phylum in dysplasia patients and increases in the Fusobacterium genus in both dysplasia and OSCC patients [26]. Thus, this study complements previous findings by showing that increases in Proteobacteria and *Fusobacteria* and decreases in Firmicutes are associated with the changes from health to oral dysplasia and to carcinogenesis. We also found progressive increases in *Burkholderiaceae* and *Pasteurellaceae* family abundance through the changes from histologically normal adjacent to tumor to low- and high-grade dysplasia. Further, we found significant lower levels of *Enterococcus cecorum*, *Fusobacterium periodonticum*, and *Prevotella melaninogenica* when moving from histologically normal to low- and high-grade dysplasia. On the other hand, *Fusobacterium canifelinum* levels were significantly higher in high grade dysplasia compared to both histologically normal adjacent and low-grade dysplasia. The loss of the former three species and gain in *Fusobacterium canifelinum* abundance can be used as potential biomarkers for oral dysplasia and OSCC. In accordance with Amer et al. [39], our study also found a decrease in species abundance from health to dysplasia and to cancer, with a lower level of similarity between species.

Next, we compared oral microbiome sampling methods, namely oral swabs and tissue biopsies processed for FFPE. Similar amounts of sequences and OTUs were obtained using both methods. Additionally, similar phyla and alpha and beta diversity trends were found with the two methods, demonstrating the feasibility of using both methods for sampling the oral microbiome for analysis. However, we observed some differences among the species recovered using these two methods, including the absence of the *Burkholderiaceae* family in the oral swab samples and different Bacteroidetes trends between the oral swabs and the unmatched tissue samples. This suggests that either (1) these microbes may be present in deeper tissues in the dysplasia specimens and the superficial swab sampling may not recover them and/or (2) that these differences may be due to comparing histologically normal tissues next to distinct lesions (i.e., comparing histologically normal adjacent to dysplasia for the swab samples and histologically normal adjacent to OSCC for tissue samples). If this is the case, then these differences might reveal possible progression changes between dysplasia and OSCC and serve as novel biomarkers for disease progression, which would be of significant clinical value from an early diagnosis perspective. In any case, it may be necessary to extract tissue samples and to compare histological normal adjacent to dysplasia against histological normal adjacent to OSCC samples to truly confirm their presence and trends. Therefore, these data indicate that oral swab sampling may be useful as an initial probe of the microbiome composition, but to determine the complete microbial signature present, tissue collection would be needed for confirmation.

Lastly, we evaluated the gene expression profile of oral cancer host cells after exposure to three species that are typically enriched in the diseased states (namely, *P. gingivalis*, *T. denticola*, and *F. nucleatum*) and one species that is decreased (*Streptococcus sanguinis*). Not only did the enriched species significantly upregulate gene processes related to cancer progression but also, they upregulated gene expression for epidermal and kerotinecyte differentiation and cornification/keratinization processes in the host cells. This finding is noteworthy as the majority of the oral cavity mucosa is non-keratinized (i.e., the lining mucosa) [89] and most dysplastic lesions of the upper aerodigestive tract are keratinized [6]. Additionally, lateral tongue and floor of the mouth dysplastic lesions (non-keratinized mucosa) have higher risk of malignant transformation [90], thus further implicating *P. gingivalis*, *T. denticola*, and *F. nucleatum* in the development of keratinized dysplasia and progression to tumor, especially in non-keratinized mucosa.

**Figure 9:**
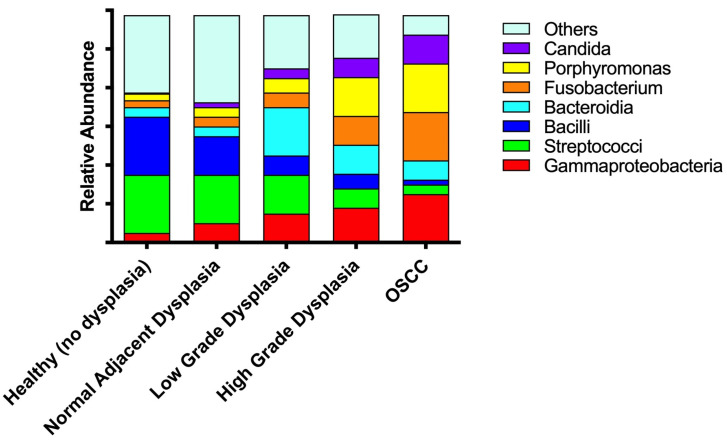
Proposed oral microbiome signature from oral health to dysplasia to OSCC.

Taken in aggregate (both literature reviews and the results presented here), we propose a theoretical description of the oral microbiome signature from health to dysplasia to OSCC (Figure 9).

In this theoretical signature the relative abundance of commensals, including *streptococci* and *bacilli* strains decreases, while there is an increase in pathogens, including *Fusobacteria*, *Porphyromonas*, *Candida*, and *Gammaproteobacteria* species in sites designated as healthy, dysplasia, or OSCC. This model also highlights the significant alterations in diversity found in our and previous reports, namely, the significant increased levels of Bacteroidia strains in low-grade dysplasia, which revert to lower levels in high-grade dysplasia and OSCC, and the disturbance in the histologically normal adjacent to dysplasia specimens, although they revealed a slight increase in diversity compared to healthy (no dysplasia) specimens.

Several clinical implications can be drawn when taking both current findings and literature reviews into consideration. These include the following: (1) the loss of commensals and expansion of pathogenic bacteria as one moves from oral health to dysplasia to OSCC is a common finding in the literature and clinical samples may reveal this pattern; (2) this pattern supports the use of potential microbial biomarkers, such as altered levels of *Neisseria baciliformis*, *Enterococcus cecorum*, *Fusobacterium periodonticum*, *Prevotella melaninogenica*, and *Fusobacterium canifelinum,* as a screening tool for states of health and disease in clinical settings; (3) in clinical settings, microbial screening with sterile cotton swabs may be potentially used instead of using biopsies; (4) potential approaches focusing on reducing pathogenic load and promoting commensal bacteria and their expansion, including preventive oral hygiene, regular periodontal maintenance visits and addressing oral biofilm related diseases may be useful to prevent and/or treat the progression from oral health to dysplasia and to OSCC; and (5) the signaling molecules/processes further enriched by pathogenic bacteria, especially the keratinization of the lining mucosa, may be useful as potential dysplasia biomarkers, therapeutic targets, and prognostic markers.

One limitation of this study was the small sample size, which could result in heterogeneity in the study. Given that only 0.1% of screened patients are diagnosed with OPMDs [67], oral dysplasia tissues are very limited and difficult to obtain. In this context, collaboration with laboratories and institutions that currently have these samples and establishment of dysplasia sample banks are necessary for future studies with larger samples sizes. Also, considering the small sample size, further stratification of the samples regarding patient smoking status was not possible, limiting the analysis for this confounding factor. However, no significant demographic differences were found (*p* = 0.5179 for oral swabs and *p* = 0.8366 for tissue samples) across groups related to smoking status. Additionally, some of the dysplasia and histologically normal adjacent tissues (especially the matched samples) were collected at the margins of OSCC, which may have contributed to the heterogeneity in the study. While it is known that some OPMD lesions can coexist at the margins of overt OSCC [91,92], assessing sequential biopsies from the same patient over time as they progress to OSCC would be the ideal approach. Another limitation of the study was that DNA was not recovered from two of the histologically normal adjacent specimens and one of the dysplasia oral swab samples. This, together with the missing bacteria (*Burkholderiaceae* family) in the oral swab samples, suggests that the oral microbiome may be more deeply embedded within the tissues and a more vigorous swabbing technique (such as a brush biopsy) may be necessary to collect enough material for analysis. Even though more than three-quarters of the samples were collected from the tongue (tissue samples) or mandibular gingiva (oral swab samples), our samples also contain other oral sites, such as soft palate and floor of the mouth. Different oral sites are known to harbor a distinct oral microbiome, forming different microbial niches in the oral cavity. For instance, the tongue has a higher density and greater diversity of microorganisms compared to other mucosal surfaces [93,94]. In this context, inclusion of different oral sites may have contributed to microbial heterogeneity in the samples. Also, this study uses two different specimen types (swab samples and tissue biopsy) and categorizes both histologically normal adjacent to dysplasia and clinically normal with no history of dysplasia as control samples, which could also contribute to heterogeneity in the study. To mitigate this, we directly compared the results, thoroughly discussed the differences found and suggested the inclusion of clinically normal (with no history of dysplasia) as a control standard for microbial analysis for future studies. Finally, time [95] and formalin fixation [96] may have affected the genetic material of the archival samples, decreasing the overall 16S rRNA library content, which could impact the overall diversity of the samples, compared to fresh samples. Yet, we were still able to generate more than 145,000 reads per sample on these FFPE archival samples, and >100,000 reads per samples is commonly recognized as sufficient for metagenomic surveys [97]. This study adds to the very limited data available in the literature on the microbiome composition of oral dysplasia and sets a precedent that future oral microbiome studies should address—whether the microbiome changes trigger gene expression changes in the host cells and tissue and vice versa.

The next steps would be to further explore metagenomics and/or meta-transcriptomics of dysplasia samples. We hope that these pilot results become the baseline for future studies, upon which larger studies demonstrating the potential causal relationships and mechanisms can be based on.

## 5. Conclusions

Our data demonstrate significant differences in the microbiome alpha and beta diversities in healthy, dysplasia, and OSCC sites, as well as increased dissimilarities among them. Moreover, we found that the Proteobacteria and Fusobacteria phyla abundance increased, concurrent with a progressive decrease in the Firmicutes phyla abundance as well as altered levels of *Enterococcus cecorum*, *Fusobacterium periodonticum*, *Prevotella melaninogenica*, and *Fusobacterium canifelinum* when moving from health to dysplasia and OSCC. Additionally, these data highlight that *P. gingivalis*, *T. denticola*, and *F. nucelatum* enrich genetic processes related to skin keratinization/cornification and cancer progression, whereas *S. sanguinis* enrich processes related to RNA processing and adhesion. These findings could represent novel biomarkers for dysplasia and OSCC disease progression.

## Figures and Tables

**Table 1:** Patient demographics—oral swab samples.

Variable	Healthy Mucosa (No Dysplasia)	Histologically Normal Adjacent/Low Grade Dysplasia (Matched)
**Age (Years ± SD)**	50.67 ± 13.58	67.50 ± 9.98
Sex		
Male	2	1
Female	2	3
**Swab Collection Location**		
Left Tongue	0	1
Right Lingual Gingiva	0	0
Left Lingual Gingiva	0	1
Mandibular Gingiva	4	2
**Dysplasia diagnosis**	-	Mild to moderate
**Smoking Status**		
Current	0	0
Past	0	0
Never	4	2
N/A	0	2

**Table 2:** Patient demographics—archival FFPE tissue specimens.

	Year	Site	Diagnosis	Age	Sex	HPV Status	Smoking Status	Alcohol Use	Other Relevant History
**Unmatched Tissue Samples**	2015	Left posterior floor of mouth	Mild dysplasia	60	M	Negative	Never smoker	One standard drink per week	History of dysplasia and squamous cell carcinoma in the left posterolateral tongue
	2010	Gingiva, between 1st and 2nd premolars	Squamous cell carcinoma in situ	82	F	Negative	n/a	n/a	History of squamous cell carcinoma in Gingiva, #7–9
	2013	Left tongue, posterior dorsal mucosal margin	Histologically normal adjacent tumor	76	M	Negative	Never smoker	2 standard drinks per week	Negative margin in a patient with left tongue squamous cell carcinoma
	2005	Left lateral tongue	Low-grade dysplasia	33	F	Negative	Never smoker	None	
	2005	Left lateral tongue	High-grade dysplasia	33					
	2012	Left lateral tongue	Mild dysplasia	60	M	Negative	Former smoker (0.25 packs per day, 1.5 pack-year)	12 standard drinks per week	History of left tongue squamous cell carcinoma in situ
	2015	Left soft palate	Moderate dysplasia	63					
	2012	Right base of tongue mucosal margin	Carcinoma in situ	54	F	Negative	Former smoker (1 pack-year, 15 pack-years, quit 25 years prior)	<1 standard drink per day	Margin in a patient with oral tongue multifocal squamous cell carcinoma
	2009	Right tongue	Moderate dysplasia	53	M	Negative	n/a	n/a	History of right lateral tongue squamous cell carcinoma and HIV+
	2017	Right tongue	Severe dysplasia	60	M	Negative	Daily tobacco chew user for 22 years	Longtime drinker	Adjacent to right tongue squamous cell carcinoma in resection specimen
	2013	Gingiva, lower left 2nd premolar region	Atypical papillary verrucous proliferation	70	F	Negative	Former smoker (1 packs per day, 24 pack-years, quit 26 years prior)	None	History of proliferative verrucous leukoplakia
	2015	Right ventral lateral tongue	Moderate dysplasia	95	F	Negative	Former smoker (0.25 packs per day, 10 pack-years, quit 35 years prior)	None	History of right tongue leukoplakia
	2014	Left tongue	Histologically normal adjacent tumor	66	F	Negative	Never smoker	None	Adjacent to left tongue squamous cell carcinoma in resection specimen
**Matched Samples**	2011	Anterior dorsal tongue	Histologically normal adjacent (margin in resection)	60	M	Negative	Former smoker (2 packs per day, 68 pack-years)	≥15 standard drinks per week	History of other recreational drug use
			Mild to moderate dysplasia (margin in resection)						
			Moderately differentiated SCC						
	2011	Left posterior ventral tongue	Histologically normal adjacent (margin in resection)	45	M	Negative	Never smoker	One standard drink per week	History of non-Hodgkin lymphoma, esophageal squamous cell carcinoma, and thyroid cancer
			Mild to moderate dysplasia (margin in resection)						
			Moderately differentiated SCC						
	2009	Anterior dorsal tongue	Hyperkeratosis, no dysplasia (margin in SCC resection)	41	M	Negative	n/a	n/a	History of proliferative verrucous leukoplakia
			Mild dysplasia (adjacent to SCC)						
			Moderately differentiated SCC						
	2009	Ventral tongue, anterior margin	Histologically normal adjacent (margin in resection)	57	M	Negative	Former smoker (60 pack-years)	One standard drink per day	
			Moderate dysplasia (margin in SCC resection)						
			Moderately differentiated SCC						
	1999	Left lateral tongue	Histologically normal adjacent (to SCCIS)	76	M	Negative	Former smoker (25 years prior)	<2 standard drink per day	
			Squamous cell carcinoma in situ with possible superficial microinvasion						
	2010	Left lateral tongue	Histologically normal adjacent (to SCCIS)	77	M	Negative	None	None	
			Mild to moderate dysplasia						
			Squamous cell carcinoma in situ						

**Table 3:** Studies evaluating the oral microbiome signature in oral dysplasia tissues.

Author/Year	Specimen Type	Method	Sample Size	Group Comparison	Microbiome	Other Remarks
Shen et al. (2023) [26]	-	Systematic Review and Metanalysis	802	-	Dysplasia—increase in phylum Bacteroidetes Dysplasia and OSCC—increase in *Fusobacterium* and decrease of *Streptococcus*	Analyzed studies presented a high risk of bias due to non-negligible heterogeneity in the type and size of the sample and inconsistent oral microbiome composition, strongly limiting the analysis. However, only 6 out of 11 analyzed studies histopathologically diagnosed their OPMDs as dysplasia, which could account for the discrepancies found.
Wright et al. (2023) [72]	Oral Swabs	16S Sequencing	90	Progressing vs. non-progressing dysplasia	Increase in *Campylobacter* in progressing dysplasia compared to non-progressing ones	No significant differences between progressing vs. non-progressing dysplasia
Herreros-Pomares et al. (2021) [73]	Tissue	16S Sequencing	10	Healthy vs. Leukoplakia + Dysplasia	Leukoplakia + dysplasia—increase in *Oribacterium* sp. *oral taxon 108*, *Campylobacter jejuni*, uncultured *Eubacterium* sp., *Tannerella*, and *Porphyromonas*	Authors have not controlled for dysplasia and mixed no dysplasia samples with mild, moderate, and severe dysplasia samples.
Sami et al. (2020) [74]	-	Reveiw	-	-	*Fusobacterium nucleatum* and *Candida* species has been associated with high-grade dysplasia and its severity	-
Gopinath et al. (2020) [41]	Whole Mouth Fluid	16S Sequencing	74	Healthy vs. Leukoplakia + Dysplasia vs. OSCC	Leukoplakia + dysplasia—increase in Bacteroidetes and decrease in Firmicutes Leuloplakia + dysplasia and OSCC—increase in Actinobacteria	Shift in bacterial communities of leukoplakia + dysplasia and oral cancer patients; no significant difference in richness and diversity
Al-Hebshi et al. (2019) [42]	-	Review	-	-	Significantly increased frequency and yeast colony counts (predominantly *Candida*) in dysplasia and OSCC; significant yeast colony increase correlated with dysplasia severity	Some of the studies did not have healthy controls, rather compared to other OPMD
Ganly et al. (2019) [27]	Saliva	16S Sequencing	38	Healthy vs. Leukoplakia + Dysplasia vs. OSCC	Leukoplakia + dysplasia—increase in *Fusobacterium* and *Veillonella* OSCC—increase in *Fusobacterium*, *Prevotella*, *Alloprevotella*; decrease in *Streptococcus*	Significantly increase in *HSP90* gene and ligands for TLRs 1, 2, and 4 along the healthy → leukoplakia/dysplasia → OSCC progression
Lee et al. (2017) [75]	Saliva	16S sequencing	376	Healthy vs. Dysplasia vs. OSCC	Significantly different levels of *Bacillus*, *Enterococcus*, *Parvimonas, Peptostreptococcus,* and *Slackia* in dysplasia compared to cancer	-
Mok et al. (2017) [76]	Oral Swabs	16S Sequencing	27	Healthy vs. Dysplasia vs. OSCC	Dysplasia—increase in *Neisseria* and *Granulicatella*; decrease in *Streptococcus*	Analysis of Molecular Variance (AMOVA) showed no significant difference between dysplasia and other groups
Amer et al. (2017) [39]	Oral Swabs	16S Sequencing	6	Healthy vs. Leukoplakia + Dysplasia	Severe dysplasia was associated with elevated levels of *Leptotrichia* spp. and *Campylobacter concisus*	-
Hebbar et al. (2013) [77]	Tissue and Oral Rinse	Periodic Acid–Schiff Staining	50	Healthy vs. Dysplasia vs. OSCC	Significant increase in yeast infection and colony number with higher dysplasia grades and OSCC	-
Spolidorio et al. (2003) [78]	Tissue	Periodic Acid–Schiff Staining	832	Healthy vs. Dysplasia	27.2% of dysplasia samples were PAS-positive; significant association of yeast infection and dysplasia	Tongue was the significant most affected site by yeast infection
McCullough et al. (2002) [79]	Tissue and Oral Rinse		223	Healthy vs. Dysplasia vs. OSCC	Dysplasia and OSCC—significantly higher frequency of oral yeast carriage and higher number of yeast (>1000 cfu/mL) than control. Correlation between dysplasia degree and yeast amount in oral cavity	
Barrett et al. (1998) [80]	Tissue	Periodic Acid–Schiff Staining	4724	Healthy vs. Dysplasia	4.7% of the biopsies contained PAS-positive fungi; significant positive association of fungal infection with moderate and severe dysplasia	Significantly higher number of males infected compared to females; 21.9% of fungi-infected dysplasia worsened in histological severity, compared to 7.6% of non-infected dysplasia
Rindum et al. (1994) [81]	Tissue	Periodic Acid–Schiff Staining and Smear Culture	153	Healthy vs. Leukoplakia/erythroleukoplakia + Dysplasia	4 *Candida albicans* strains were found in moderate and severe dysplasia, but none on mild dysplasia	-
Krogh et al. (1987) [70]	Swab over biopsy	Yeast culture	12	Healthy vs. leukoplakia/erythroleukoplakia +Dysplasia	4 different strains of *Candida albicans*, one strain of *Candida parapsilosis* found in dysplasia samples	Samples positive for dysplasia showed yeast strains with lower nitrosation potential compared to the ones on samples negative for dysplasia (no statistical analysis)

## Data Availability

The datasets presented in this study can be found in online repositories. The names of the repository/repositories and accession number(s) can be found below: https://doi.org/10.6084/m9.figshare.23315222.v1 (accessed on 1 July 2023).

## Supplementary Materials

The following supporting information can be downloaded at https://www.mdpi.com/article/10.3390/microorganisms11092250/s1, Figure S1: observed species in the histologically normal adjacent oral swab samples are not significant different than histologically normal adjacent matched and unmatched tissue samples. Figure S2: *Pasteurellaceae* and *Burkoholdericeae* families were significantly increased in low- and high-grade dysplasia compared to histologically normal samples, respectively. Figure S3: *Streptococci* genus is significantly reduced in the histologically normal adjacent to dysplasia specimens compared to healthy (no dysplasia) oral swab samples.
