# Supplementary figures and images for: Specific Oral Microbial Differences in Proteobacteria and Bacteroidetes Are Associated with Distinct Sites When Moving from Healthy Mucosa to Oral Dysplasia—A Microbiome and Gene Profiling Study and Focused Review
Source: Microorganisms. 2023 Sep 7;11(9):2250. doi: 10.3390/microorganisms11092250 (PMC10534919; doi:10.3390/microorganisms11092250)

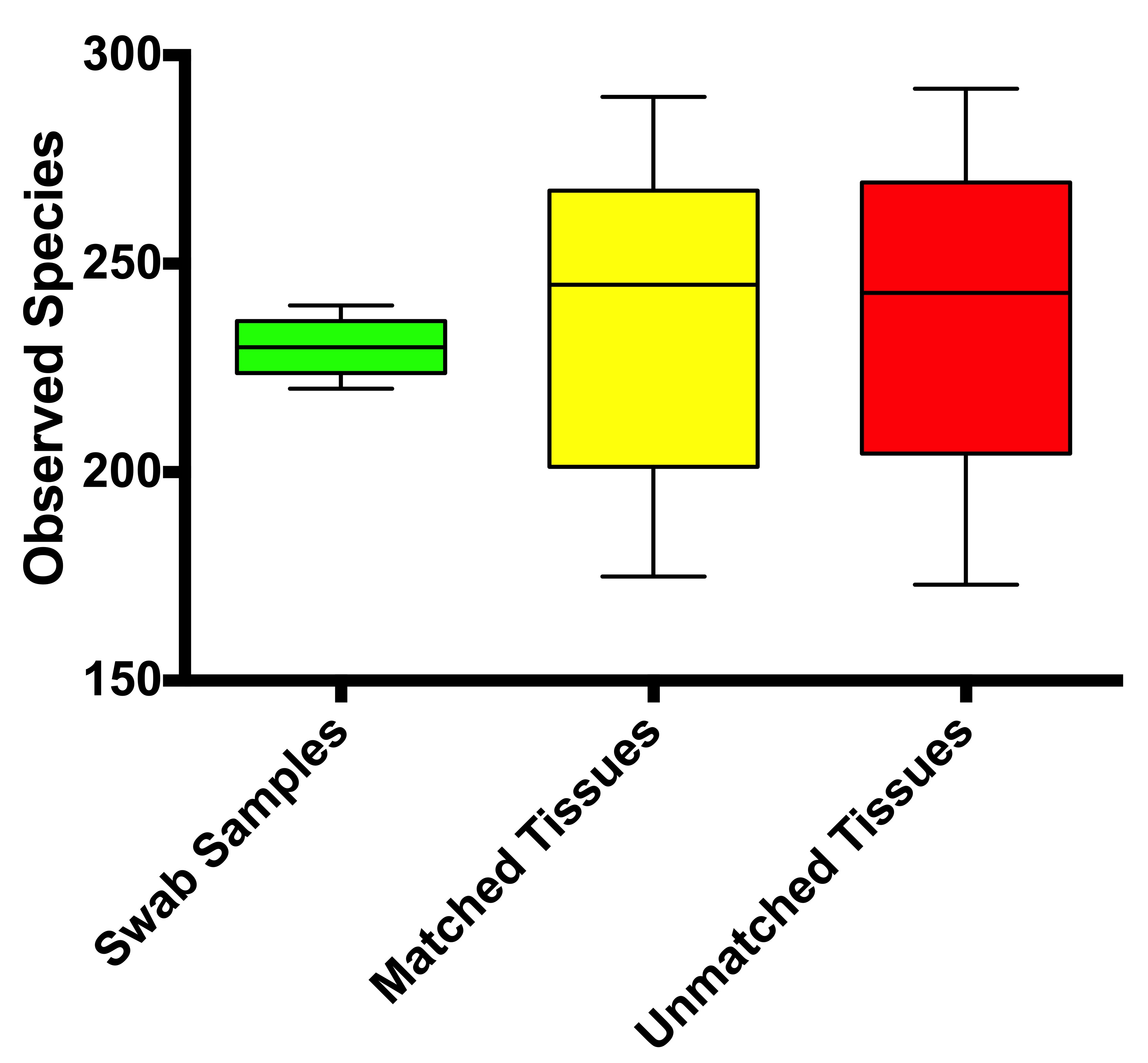

Supplement: Supplementary file 1 [file microorganisms-11-02250-s001.zip › Supplementary material/Figure S1.jpg]

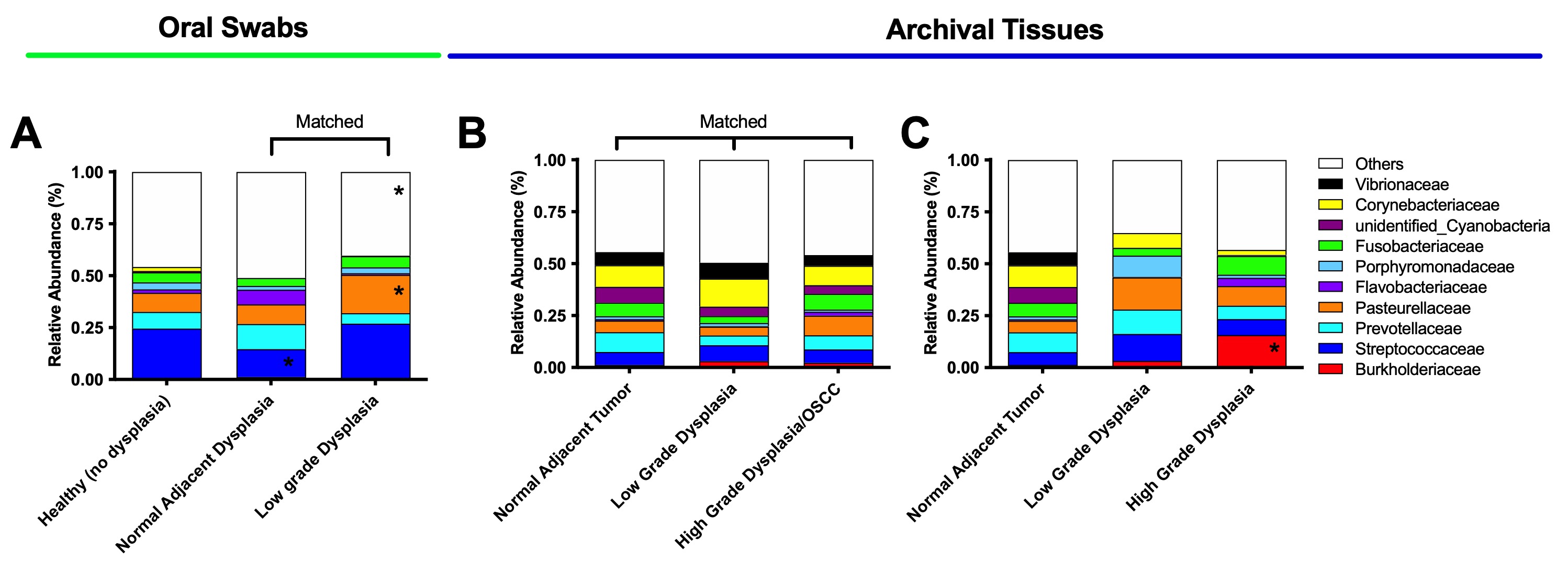

Supplement: Supplementary file 1 [file microorganisms-11-02250-s001.zip › Supplementary material/Figure S2.jpg]

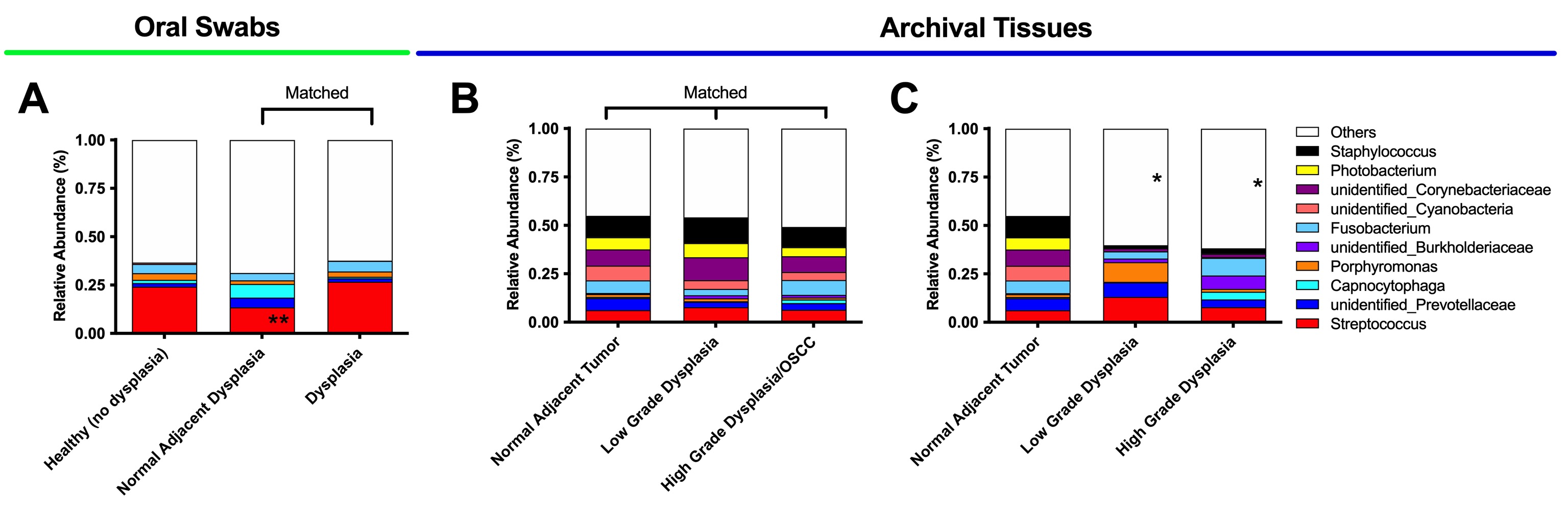

Supplement: Supplementary file 1 [file microorganisms-11-02250-s001.zip › Supplementary material/Figure S3.jpg]
